# Supplementary material for: Efficacy of Colchicine in the Treatment of COVID-19 Patients: A Systematic Review and Meta-Analysis
Source: J Clin Med. 2022 May 6;11(9):2615. doi: 10.3390/jcm11092615 (PMC9105993; doi:10.3390/jcm11092615)

Efficacy of colchicine in the treatment of COVID-19 patients: a systematic review and meta-analysis

Supplementary contents

- **Supplementary Table S1:** Search strategy for each engine.
- **Supplementary Table S2:** Ongoing randomized controlled trials.
- **Supplementary Table S3:** GRADE summary of findings table.
- **Supplementary Figure S1:** Risk of bias assessment of included trials.
- **Supplementary Figure S2:** Sensitivity analyses of the effects of colchicine vs standard of care on mortality.

Supplementary Table S1: Search strategy for each engine.

Engine	Search strategy	Results
**PubMed**	**#1:** COVID-19[MH] OR COVID 19[MH] OR COVID-19 Virus Disease[MH] OR COVID 19 Virus Disease[MH] OR COVID-19 Virus Diseases[MH] OR Disease, COVID-19 Virus[MH] OR Virus Disease, COVID-19[MH] OR COVID-19 Virus Infection[MH] OR COVID 19 Virus Infection[MH] OR COVID-19 Virus Infections[MH] OR Infection, COVID-19 Virus[MH] OR Virus Infection, COVID-19[MH] OR 2019-nCoV Infection[MH] OR 2019 nCoV Infection[MH] OR 2019-nCoV Infections[MH] OR Infection, 2019-nCoV[MH] OR Coronavirus Disease-19[MH] OR Coronavirus Disease 19[MH] OR 2019 Novel Coronavirus Disease[MH] OR 2019 Novel Coronavirus Infection[MH] OR 2019-nCoV Disease[MH] OR 2019 nCoV Disease[MH] OR 2019-nCoV Diseases[MH] OR Disease, 2019-nCoV[MH] OR COVID19[MH] OR Coronavirus Disease 2019[MH] OR Disease 2019, Coronavirus[MH] OR SARS Coronavirus 2 Infection[MH] OR SARS-CoV-2 Infection[MH] OR Infection, SARS-CoV-2[MH] OR SARS CoV 2 Infection[MH] OR SARS-CoV-2 Infections[MH] OR COVID-19 Pandemic[MH] OR COVID 19 Pandemic[MH] OR COVID-19 Pandemics[MH] OR Pandemic, COVID-19[MH] OR SARS-CoV-2[MH] OR Coronavirus Disease 2019 Virus[MH] OR 2019 Novel Coronavirus[MH] OR 2019 Novel Coronaviruses[MH] OR Coronavirus, 2019 Novel[MH] OR Novel Coronavirus, 2019[MH] OR Wuhan Seafood Market Pneumonia Virus[MH] OR SARS-CoV-2 Virus[MH] OR SARS CoV 2 Virus[MH] OR SARS-CoV-2 Viruses[MH] OR Virus, SARS-CoV-2[MH] OR 2019-nCoV[MH] OR COVID-19 Virus[MH] OR COVID 19 Virus[MH] OR COVID-19 Viruses[MH] OR Virus, COVID-19[MH] OR Wuhan Coronavirus[MH] OR Coronavirus, Wuhan[MH] OR SARS Coronavirus 2[MH] OR Coronavirus 2, SARS[MH] OR Severe Acute Respiratory Syndrome Coronavirus 2[MH] **OR** “COVID-19”[TIAB] OR “COVID 19”[TIAB] OR “COVID-19 Virus Disease*”[TIAB] OR “COVID 19 Virus Disease*”[TIAB] OR “COVID-19 Virus Infection*”[TIAB] OR “COVID 19 Virus Infection*”[TIAB] OR “2019-nCoV Infection*”[TIAB] OR “2019 nCoV Infection*”[TIAB] OR “Coronavirus Disease-19”[TIAB] OR “Coronavirus Disease 19”[TIAB] OR “2019 Novel Coronavirus Disease”[TIAB] OR “2019 Novel Coronavirus Infection”[TIAB] OR “2019-nCoV Disease*”[TIAB] OR “2019 nCoV Disease*”[TIAB] OR COVID19[TIAB] OR “Coronavirus Disease 2019”[TIAB] OR "SARS Coronavirus 2 Infection”[TIAB] OR “SARS-CoV-2 Infection*”[TIAB] OR “SARS CoV 2 Infection*”[TIAB] OR “COVID-19 Pandemic*”[TIAB] OR “COVID 19 Pandemic*”[TIAB] OR “COVID 2019”[TIAB] OR “Sars Coronavirus 2 infection*”[TIAB] OR “sarscov2 disease*”[TIAB] OR “sarscov2 infection*”[TIAB] OR “sars-cov2 infection*”[TIAB] OR “sars-cov2 disease*”[TIAB] OR “wuhan coronavirus disease*”[TIAB] OR “wuhan coronavirus infection*”[TIAB] OR “ncov 2019 disease*”[TIAB] OR “ncov 2019 infection*”[TIAB] OR “novel coronavirus 2019 disease*”[TIAB] OR “novel coronavirus 2019 infection*”[TIAB] OR “novel coronavirus disease* 2019”[TIAB] OR “novel coronavirus infection* 2019”[TIAB] OR SARS-CoV-2[TIAB] OR “Coronavirus Disease 2019 Virus”[TIAB] OR “2019 Novel Coronavirus*”[TIAB] OR “Wuhan Seafood Market Pneumonia Virus”[TIAB] OR “SARS-CoV-2 Virus*”[TIAB] OR “SARS CoV 2 Virus*”[TIAB] OR 2019-nCoV[TIAB] OR “COVID-19 Virus*”[TIAB] OR “COVID 19 Virus*”[TIAB] OR “Wuhan Coronavirus”[TIAB] OR “SARS Coronavirus 2”[TIAB] OR “Severe Acute Respiratory Syndrome Coronavirus 2”[TIAB] OR “sars-related coronavirus”[TIAB] OR “sars-like cov”[TIAB] OR “sars-like coronavirus”[TIAB] OR “sars-related cov”[TIAB] OR “sarsr-cov”[TIAB] OR “severe acute respiratory syndrome-like coronavirus”[TIAB] OR “severe acute respiratory syndrome-related coronavirus”[TIAB] OR “sars coronavirus”[TIAB] OR hcov-sars[TIAB] OR “human sars coronavirus”[TIAB] OR “sars cov”[TIAB] OR “sars associated coronavirus”[TIAB] OR “sars virus”[TIAB] OR sars-cov[TIAB] OR “sars-associated coronavirus”[TIAB] OR “severe acute respiratory syndrome coronavirus”[TIAB] OR	107

	“severe acute respiratory syndrome virus”[TIAB] OR “Coronavirus disease*”[TIAB] OR “severe acute respiratory virus coronavirus 2”[TIAB] OR “coronavirus pandemic”[TIAB] OR Coronavirus*[TIAB] OR “SARS-COV2 pandemic”[TIAB] OR “SARS-COV-2 pandemic”[TIAB] OR “SARS COV2 pandemic”[TIAB] **OR** “COVID-19”[OT] OR “COVID 19”[OT] OR “COVID-19 Virus Disease*”[OT] OR “COVID 19 Virus Disease*”[OT] OR “COVID-19 Virus Infection*”[OT] OR “COVID 19 Virus Infection*”[OT] OR “2019-nCoV Infection*”[OT] OR “2019 nCoV Infection*”[OT] OR “Coronavirus Disease-19”[OT] OR “Coronavirus Disease 19”[OT] OR “2019 Novel Coronavirus Disease”[OT] OR “2019 Novel Coronavirus Infection”[OT] OR “2019-nCoV Disease*”[OT] OR “2019 nCoV Disease*”[OT] OR COVID19[OT] OR “Coronavirus Disease 2019”[OT] OR "SARS Coronavirus 2 Infection”[OT] OR “SARS-CoV-2 Infection*”[OT] OR “SARS CoV 2 Infection*”[OT] OR “COVID-19 Pandemic*”[OT] OR “COVID 19 Pandemic*”[OT] OR “COVID 2019”[OT] OR “Sars Coronavirus 2 infection*”[OT] OR “sarscov2 disease*”[OT] OR “sarscov2 infection*”[OT] OR “sars-cov2 infection*”[OT] OR “sars-cov2 disease*”[OT] OR “wuhan coronavirus disease*”[OT] OR “wuhan coronavirus infection*”[OT] OR “ncov 2019 disease*”[OT] OR “ncov 2019 infection*”[OT] OR “novel coronavirus 2019 disease*”[OT] OR “novel coronavirus 2019 infection*”[OT] OR “novel coronavirus disease* 2019”[OT] OR “novel coronavirus infection* 2019”[OT] OR SARS-CoV-2[OT] OR “Coronavirus Disease 2019 Virus”[OT] OR “2019 Novel Coronavirus*”[OT] OR “Wuhan Seafood Market Pneumonia Virus”[OT] OR “SARS-CoV-2 Virus*”[OT] OR “SARS CoV 2 Virus*”[OT] OR 2019-nCoV[OT] OR “COVID-19 Virus*”[OT] OR “COVID 19 Virus*”[OT] OR “Wuhan Coronavirus”[OT] OR “SARS Coronavirus 2”[OT] OR “Severe Acute Respiratory Syndrome Coronavirus 2”[OT] OR “sars-related coronavirus”[OT] OR “sars-like cov”[OT] OR “sars-like coronavirus”[OT] OR “sars-related cov”[OT] OR “sarsr-cov”[OT] OR “severe acute respiratory syndrome-like coronavirus”[OT] OR “severe acute respiratory syndrome-related coronavirus”[OT] OR “sars coronavirus”[OT] OR hcov-sars[OT] OR “human sars coronavirus”[OT] OR “sars cov”[OT] OR “sars associated coronavirus”[OT] OR “sars virus”[OT] OR sars-cov[OT] OR “sars-associated coronavirus”[OT] OR “severe acute respiratory syndrome coronavirus”[OT] OR “severe acute respiratory syndrome virus”[OT] OR “Coronavirus disease*”[OT] OR “severe acute respiratory virus coronavirus 2”[OT] OR “coronavirus pandemic”[OT] OR Coronavirus*[OT] OR “SARS-COV2 pandemic”[OT] OR “SARS-COV-2 pandemic”[OT] OR “SARS COV2 pandemic”[OT]<br><br>**#2:** Colchicine[MH] OR Colchicin*[TIAB] OR Colchicin*[OT]<br><br>**#3:** #1 AND #2	
**Scopus**	#1: TITLE-ABS-KEY(“COVID-19” OR “COVID 19” OR “COVID-19 Virus Disease*” OR “COVID 19 Virus Disease*” OR “COVID-19 Virus Infection*” OR “COVID 19 Virus Infection*” OR “2019-nCoV Infection*” OR “2019 nCoV Infection*” OR “Coronavirus Disease-19” OR “Coronavirus Disease 19” OR “2019 Novel Coronavirus Disease” OR “2019 Novel Coronavirus Infection” OR “2019-nCoV Disease*” OR “2019 nCoV Disease*” OR COVID19 OR “Coronavirus Disease 2019” OR "SARS Coronavirus 2 Infection” OR “SARS-CoV-2 Infection*” OR “SARS CoV 2 Infection*” OR “COVID-19 Pandemic*” OR “COVID 19 Pandemic*” OR “COVID 2019” OR “Sars Coronavirus 2 infection*” OR “sarscov2 disease*” OR “sarscov2 infection*” OR “sars-cov2 infection*” OR “sars-cov2 disease*” OR “wuhan coronavirus disease*” OR “wuhan coronavirus infection*” OR “ncov 2019 disease*” OR “ncov 2019 infection*” OR “novel coronavirus 2019 disease*” OR “novel coronavirus 2019 infection*” OR “novel coronavirus disease* 2019” OR “novel coronavirus infection* 2019” OR SARS-CoV-2 OR “Coronavirus Disease 2019 Virus” OR “2019 Novel Coronavirus*” OR “Wuhan Seafood Market Pneumonia	217

	Virus” OR “SARS-CoV-2 Virus*” OR “SARS CoV 2 Virus*” OR 2019-nCoV OR “COVID-19 Virus*” OR “COVID 19 Virus*” OR “Wuhan Coronavirus” OR “SARS Coronavirus 2” OR “Severe Acute Respiratory Syndrome Coronavirus 2” OR “sars-related coronavirus” OR “sars-like cov” OR “sars-like coronavirus” OR “sars-related cov” OR “sarsr-cov” OR “severe acute respiratory syndrome-like coronavirus” OR “severe acute respiratory syndrome-related coronavirus” OR “sars coronavirus” OR hcov-sars OR “human sars coronavirus” OR “sars cov” OR “sars associated coronavirus” OR “sars virus” OR sars-cov OR “sars-associated coronavirus” OR “severe acute respiratory syndrome coronavirus” OR “severe acute respiratory syndrome virus” OR “Coronavirus disease*” OR “severe acute respiratory virus coronavirus 2” OR “coronavirus pandemic” OR Coronavirus* OR “SARS-COV2 pandemic” OR “SARS-COV-2 pandemic” OR “SARS COV2 pandemic”)<br><br>**#2:** TITLE-ABS-KEY(Colchicin*)<br><br>**#3:** #1 AND #2	
**Wos/Core Collection**	**#1:** TS=(“COVID-19” OR “COVID 19” OR “COVID-19 Virus Disease*” OR “COVID 19 Virus Disease*” OR “COVID-19 Virus Infection*” OR “COVID 19 Virus Infection*” OR “2019-nCoV Infection*” OR “2019 nCoV Infection*” OR “Coronavirus Disease-19” OR “Coronavirus Disease 19” OR “2019 Novel Coronavirus Disease” OR “2019 Novel Coronavirus Infection” OR “2019-nCoV Disease*” OR “2019 nCoV Disease*” OR COVID19 OR “Coronavirus Disease 2019” OR "SARS Coronavirus 2 Infection” OR “SARS-CoV-2 Infection*” OR “SARS CoV 2 Infection*” OR “COVID-19 Pandemic*” OR “COVID 19 Pandemic*” OR “COVID 2019” OR “Sars Coronavirus 2 infection*” OR “sarscov2 disease*” OR “sarscov2 infection*” OR “sars-cov2 infection*” OR “sars-cov2 disease*” OR “wuhan coronavirus disease*” OR “wuhan coronavirus infection*” OR “ncov 2019 disease*” OR “ncov 2019 infection*” OR “novel coronavirus 2019 disease*” OR “novel coronavirus 2019 infection*” OR “novel coronavirus disease* 2019” OR “novel coronavirus infection* 2019” OR SARS-CoV-2 OR “Coronavirus Disease 2019 Virus” OR “2019 Novel Coronavirus*” OR “Wuhan Seafood Market Pneumonia Virus” OR “SARS-CoV-2 Virus*” OR “SARS CoV 2 Virus*” OR 2019-nCoV OR “COVID-19 Virus*” OR “COVID 19 Virus*” OR “Wuhan Coronavirus” OR “SARS Coronavirus 2” OR “Severe Acute Respiratory Syndrome Coronavirus 2” OR “sars-related coronavirus” OR “sars-like cov” OR “sars-like coronavirus” OR “sars-related cov” OR “sarsr-cov” OR “severe acute respiratory syndrome-like coronavirus” OR “severe acute respiratory syndrome-related coronavirus” OR “sars coronavirus” OR hcov-sars OR “human sars coronavirus” OR “sars cov” OR “sars associated coronavirus” OR “sars virus” OR sars-cov OR “sars-associated coronavirus” OR “severe acute respiratory syndrome coronavirus” OR “severe acute respiratory syndrome virus” OR “Coronavirus disease*” OR “severe acute respiratory virus coronavirus 2” OR “coronavirus pandemic” OR Coronavirus* OR “SARS-COV2 pandemic” OR “SARS-COV-2 pandemic” OR “SARS COV2 pandemic”)<br><br>**#2:** TS=colchicin*<br><br>**#3:** #1 AND #2	74
**Ovid/Medline**	**#1:** COVID-19.mp. or COVID-19/<br>**#2:** COVID-19/ or COVID.mp.<br>**#3:** #1 OR #2<br><br>**#4:** Colchicine.mp. or Colchicine/<br><br>**#5:** #3 AND #4	103

**Embase**	**#1:** 'coronavirus disease 2019'/exp OR 'coronavirus disease 2019':ti,ab OR '2019 novel coronavirus disease':tn,ti,ab OR '2019 novel coronavirus infection':tn,ti,ab OR '2019-ncov disease':tn,ti,ab OR '2019-ncov infection':tn,ti,ab OR 'covid':tn,ti,ab OR 'covid 19':tn,ti,ab OR 'covid 2019':tn,ti,ab OR 'covid-19':tn,ti,ab OR 'covid19':tn,ti,ab OR 'sars coronavirus 2 infection':tn,ti,ab OR 'sars-cov-2 disease':tn,ti,ab OR 'sars-cov-2 infection':tn,ti,ab OR 'sars-cov2 disease':tn,ti,ab OR 'sars-cov2 infection':tn,ti,ab OR 'sarscov2 disease':tn,ti,ab OR 'sarscov2 infection':tn,ti,ab OR 'wuhan coronavirus disease':tn,ti,ab OR 'wuhan coronavirus infection':tn,ti,ab OR 'coronavirus disease 2019':tn,ti,ab OR 'ncov 2019 disease':tn,ti,ab OR 'ncov 2019 infection':tn,ti,ab OR 'novel coronavirus 2019 disease':tn,ti,ab OR 'novel coronavirus 2019 infection':tn,ti,ab OR 'novel coronavirus disease 2019':tn,ti,ab OR 'novel coronavirus infection 2019':tn,ti,ab OR 'coronavirus disease 2019'/exp/'adverse device effect'<br><br>**#2:** 'colchicine'/exp OR colchicine:ti,ab OR '7 acetamido 6, 7 dihydro 1, 2, 3, 10 tetramethoxybenzo [a] heptalen 9 (5h) one':tn,ti,ab OR 'aqua colchin':tn,ti,ab OR 'colchichine':tn,ti,ab OR 'colchicin':tn,ti,ab OR 'colchicina':tn,ti,ab OR 'colchicine':tn,ti,ab OR 'colchicine capsules':tn,ti,ab OR 'colchicine houde':tn,ti,ab OR 'colchicine sodium':tn,ti,ab OR 'colchicum-dispert':tn,ti,ab OR 'colchily':tn,ti,ab OR 'colchimedio':tn,ti,ab OR 'colchiquim':tn,ti,ab OR 'colchisol':tn,ti,ab OR 'colchysat':tn,ti,ab OR 'colcine':tn,ti,ab OR 'colcrys':tn,ti,ab OR 'colctab':tn,ti,ab OR 'colgout':tn,ti,ab OR 'colrefuz':tn,ti,ab OR 'gloperba':tn,ti,ab OR 'goutichine':tn,ti,ab OR 'goutnil':tn,ti,ab OR 'kolkicin':tn,ti,ab OR 'kolkisin':tn,ti,ab OR 'mitigare':tn,ti,ab OR 'mpc 004':tn,ti,ab OR 'mpc004':tn,ti,ab OR 'n (1, 2, 3, 10 tetramethoxy 9 oxo 5, 6, 7, 9 tetrahydrobenzo [a] heptalen 7 yl) acetamide':tn,ti,ab OR 'n (1, 2, 3, 10 tetramethoxy 9 oxo 6, 7 dihydro 5h benzo [a] heptalen 7 yl) acetamide':tn,ti,ab OR 'n (5, 6, 7, 9 tetrahydro 1, 2, 3, 10 tetramethoxy 9 oxobenzo [a] heptalen 7 yl) acetamide':tn,ti,ab OR 'n acetyl trimethylcolchicinic acid methyl ether':tn,ti,ab OR 'nsc 757':tn,ti,ab OR 'tolchicine':tn,ti,ab OR 'colchicine'/exp/'adverse device effect'<br><br>**#3:** #1 AND #2	253
**Cochrane CENTRAL**	**#1:** MeSH descriptor: [COVID-19] explode all trees<br>**#2:** ("COVID-19" OR "COVID 19" OR "COVID-19 Virus Disease*" OR "COVID 19 Virus Disease*" OR "COVID-19 Virus Infection*" OR "COVID 19 Virus Infection*" OR "2019 nCoV Infection*" OR "Coronavirus Disease-19" OR "Coronavirus Disease 19" OR "2019 Novel Coronavirus Disease" OR "2019 Novel Coronavirus Infection" OR "2019 nCoV Disease*" OR "COVID19" OR "Coronavirus Disease 2019" OR "SARS Coronavirus 2 Infection" OR "SARS-CoV-2 Infection*" OR "SARS CoV 2 Infection*" OR "COVID-19 Pandemic*" OR "COVID 19 Pandemic*" OR "COVID 2019" OR "Sars Coronavirus 2 infection*" OR "sarscov2 disease*" OR "sarscov2 infection*" OR "sars-cov2 infection*" OR "sars-cov2 disease*" OR "wuhan coronavirus disease*" OR "wuhan coronavirus infection*" OR "ncov 2019 disease*" OR "ncov 2019 infection*" OR "novel coronavirus 2019 disease*" OR "novel coronavirus 2019 infection*" OR "novel coronavirus disease* 2019" OR "novel coronavirus infection* 2019" OR "SARS-CoV-2" OR "Coronavirus Disease 2019 Virus" OR "2019 Novel Coronavirus*" OR "Wuhan Seafood Market Pneumonia Virus" OR "SARS-CoV-2 Virus*" OR "SARS CoV 2 Virus*" OR "COVID-19 Virus*" OR "COVID 19 Virus*" OR "Wuhan Coronavirus" OR "SARS Coronavirus 2" OR "Severe Acute Respiratory Syndrome Coronavirus 2" OR "sars-related coronavirus" OR "sars-like cov" OR "sars-like coronavirus" OR "sarsr-cov" OR "severe acute respiratory syndrome-like coronavirus" OR "severe acute respiratory syndrome-related coronavirus" OR "sars coronavirus" OR "hcov-sars" OR "human sars coronavirus" OR "sars cov" OR "sars associated coronavirus" OR "sars virus" OR "sars-cov" OR "sars-associated coronavirus" OR "severe acute respiratory syndrome coronavirus" OR "severe acute respiratory syndrome virus" OR "Coronavirus disease*" OR "severe acute respiratory virus coronavirus 2" OR "coronavirus	43

	pandemic" OR "Coronavirus*" OR "SARS-COV2 pandemic" OR "SARS-COV-2 pandemic" OR "SARS COV2 pandemic"):ti,ab,kw<br><br>**#3:** #1 OR #2<br><br>**#4:** MeSH descriptor: [Colchicine] explode all trees<br>**#5:** ("colchicine"):ti,ab,kw<br>**#6:** #4 OR #5<br><br>**#7:** #3 AND #6	

Supplementary Table S2: Ongoing randomized controlled trials (as of July 30, 2021).

Trial ID	Trial title	Experimental arm	Control arm	Country	Estimated enrollment	Estimated study completion date
NCT04375202	Colchicine in COVID-19: a Pilot Study (COLVID-19)	Colchicine tablets containing 1 mg, administered 0.5 mg po every 8 hours x 30 days	Current care	Italy	308 patients	October 31, 2021
NCT04355143	Colchicine to Reduce Cardiac Injury in COVID-19 (COLHEART-19) (COLHEART-19)	Colchicine 0.6 mg po BID x 30 days plus current care per UCLA treating physicians	Current care per UCLA physicians alone	United States	150 patients	April 25, 2021
NCT04360980	The Effects of Standard Protocol with or Without Colchicine in Covid-19 Infection	Colchicine Tablets<br>1.5 mg loading then 0.5 mg BID	Standard treatment<br>40 RT-PCR positive COVID-19 patients without hypoxemia receiving vitamin C 3grams daily, 400 mg Tiamine, Selenium, Omega-3 500 mg daily, Vit A, Vit D, Azithromycine, Ceftriaxone, Kaletra 400 BID 10 days	Iran	80 patients	November 30, 2021
NCT04756128	Impact of Colchicine and Lowdose Naltrexone on COVID-19	Colchicine-containing treatment arm will receive colchicine 0.6 mg twice daily for up to 28 days.	Patients in this arm will receive the investigating institution's current standard of care for patients with COVID-19.	United States	164 patients	December 31, 2021
NCT04492358	Treatment for Moderate/Severe COVID-19 in a Fragile and Vulnerable Population, Admitted to a Geriatric Hospital Unit or in a Transicional Care Center	Prednisone 60 mg/day, in a single dose, for 3 days and Colchicine ideal dose of 0.3 mg/kg/day	Standard treatment	Spain	144 patients	October 2021
NCT04818489	Colchicine and Post-COVID-19 Pulmonary Fibrosis	Colchicine 0.5 mg (2 tablets: 1 mg) twice per day as a loading dose, followed by one tablet 0.5 twice per day for three weeks in addition to the standard protocol	Local standard protocol for COVID19	Egypt	250 patients	July 10, 2021
NCT04472611	Colchicine/Statins for the Prevention of COVID-19 Complications (COLSTAT) Trial (COLSTAT)	Rosuvastatin 40mg daily and Colchicine 0.6mg twice for 3 days	Standard of care treatment	United States	466 patients	August 1, 2021

		and then 0.6mg daily during hospitalization				
NCT04516941	CorONa Virus edoxabaN ColchicinE (CONVINCE) COVID-19	Colchicine at 0.5 mg per os (PO) twice daily for the first 3 days and then once daily from randomization to day 14 (+/-3) days. Treatment could be continued to day 25 (+3/-3 days).	No intervention	Belgium, Italy, Spain, Switzerland	420 patients	June 30, 2023
NCT04724629	Survival TRial Using CytoKines in COVID-19 (STRUCK Trial)	Patients will receive study medication colchicine 0.5 mg every 8 hours for 3 days (PO), followed by 4 weeks (+/-7 days) 0.5 mg twice daily. If a dose is missed, it should not be replaced.	Standard treatment, supplementation of O2 ventilation + standard treatment of the institution, which may include Dexamethasone according to the institutional protocol.	Brazil	60 patients	July 30, 2021
NCT04416334	Preemptive therapy with colchicine in patients older than 60 years with high risk of severe pneumoniae due to coronavirus (COLCHI-COVID)	Colchicine 0.5 mg orally (PO) twice daily for the first 3 days and then once daily for the last 18 days plus symptomatic treatment (paracetamol)	Symptomatic treatment (paracetamol or best symptomatic treatment based on doctor recommendations)	Spain	954 patients	December 31,2021
NCT04403243	COLchicine Versus Ruxolitinib and Secukinumab In Open Prospective Randomized Trial	30 Patients with mild and severe COVID 19 Patients will get investigated therapy for ten days. Patients will be follow-up during 45 days after randomization	30 patients Patients with mild and severe COVID 19 Patients will get investigated therapy for ten days. Patients will be follow-up during 45 days after randomization	Russian Federation	70 patients	August 23, 2020
NCT04322565	Colchicine Counteracting Inflammation in COVID-19 Pneumonia (ColCOVID-19)	Colchicine 1mg (or 0.5 mg in CKD)/day + standard of care for COVID-19 pneumonia	Standard of care	Italy	310 patients	December 21, 2020
NCT04367168	Colchicine Twice Daily During 10 Days as an Option for the Treatment of Symptoms Induced by Inflammation in Patients With Mild and Severe Coronavirus Disease (ColchiVID)	Colchicine 1mg, 1 ½ pill in day 1 and ½ pill BID during 10 days	Placebo, 1 ½ pill in day 1 and ½ pill BID during 10 days	Mexico	174 patients	April 27, 2021
NCT04539873	Impact of Colchicine in Hospitalized Colombian Patients With COVID-19 ((COLCOVID19))	Colchicine 1,5 orally on the first day (initially two pills of 0,5 mg and 0.5 mg at 2 hours), followed	Standard treatment	Colombia	128 patients	December 15, 2021

		by 0.5 mg every 12 hours on days 2 to 7, and continuing with 0.5 mg per day until completing 14 ± 1 days				
NCT04363437	COlchicine in Moderate-severe Hospitalized Patients Before ARDS to Treat COVID-19 (COMBATCOVID19)	Colchicine starting dose of 1.2 mg followed, by 0.6mg after 2 hours if they do not have significant gastrointestinal symptoms, on day 1. After that, they will take colchicine 0.6mg twice a day for 14 days or until discharged or release from the hospital.	Usual care COVID19 treatment and will not receive colchicine.	United States	70 patients	June 14, 2020
NCT04381936	Randomised Evaluation of COVID-19 Therapy	Colchicine<br>First (main) randomisation part A. 1 mg after randomisation followed by 500mcg 12 hours later and then 500 mcg twice daily by mouth or nasogastric tube for 10 days in total, for men ≥18 years old and women ≥55 years old only	Patient receives usual hospital care.	Indonesia, Nepal, United Kingdom	45000 patients	December 2021
NCT04324463	Anti-Coronavirus Therapies to Prevent Progression of Coronavirus Disease 2019 (COVID-19) Trial	Outpatients: 0.6 mg twice daily for 3 days, then 0.6 mg once daily for 25 days (total 28 days).<br>Inpatients: 1.2 mg followed by 0.6 mg 2 hours later, then 0.6 mg twice daily for 28 days.	Outpatients and Inpatients: No constraints for treating physicians on the therapies within the standard of care arm. All key co-interventions will be documented.	Brazil, Canada Colombia, Ecuador, Egypt, India, Pakistan, Philippines, Russian Federation, Saudi Arabia, United Arab Emirates	4000 patients	April 30, 2022

Supplemental Table S3: GRADE Summary of findings of the effect of Colchicine vs. Standard of care in the treatment of COVID-19 patients.

Certainty assessment							№ of patients		Effect		Certainty	Importance
№ of studies	Study design	Risk of bias	Inconsistency	Indirectness	Imprecision	Other considerations	Colchicine	Standard of care	Relative (95% CI)	Absolute (95% CI)		
**Mortality**												
3	randomised trials	very serious [a]	not serious [b]	not serious	very serious [c]	none	1174/5683 (20.7%)	1194/5797 (20.6%)	**RR 1.01** (0.86 to 1.17)	**2 more per 1,000** (from 29 fewer to 35 more)	⨁◯◯◯ VERY LOW	
**Mortality**												
4	observational studies	very serious [a]	serious [d]	not serious	not serious	none	44/259 (17.0%)	146/322 (45.3%)	**RR 0.45** (0.26 to 0.78)	**249 fewer per 1,000** (from 336 fewer to 100 fewer)	⨁◯◯◯ VERY LOW	
**Length of hospitalization**												
2	randomised trials	very serious [a]	not serious [b]	not serious	very serious [e]	none	73	67	-	MD **2.25 days lower** (9.34 lower to 4.84 higher)	⨁◯◯◯ VERY LOW	

CI: Confidence interval; **RR:** Risk ratio; **MD:** Mean difference

Explanations

a. There is a high risk of bias in at least one of the studies for this outcome.
b. Low heterogeneity but not significantly effect
c. No significant effect RR 1.01; 95%CI 0.86 to 1.17
d. Moderate heterogeneity=22%
e. No significant effect -2.25; 95%CI -9.34 to 4.84

Supplemental Figure S1: Risk of bias assessment of included trials.

Unique ID	D1	D2	D3	D4	D5	Overall
Deftereos et al. 2020	+	-	+	+	!	-
Lopes et al. 2020	+	!	-	+	!	-
Diaz, 2021	+	+	+	+	+	+
Horby et al, 2021	+	!	!	+	!	!
Absalon, 2021	+	+	+	+	+	+

+	Low risk
!	Some concerns
-	High risk

D1	Randomisation process
D2	Deviations from the intended interventions
D3	Missing outcome data
D4	Measurement of the outcome
D5	Selection of the reported result

Supplemental Figure S2: Sensitivity analyses of the effects of colchicine vs standard of care on mortality.

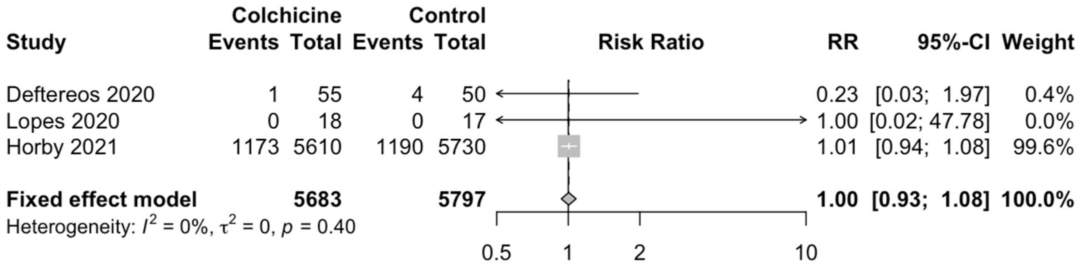

Supplement: Supplementary file 1 [file jcm-11-02615-s001.zip › jcm-1655418-supplementary.pdf]
